# Optomechanically induced transparency in the presence of an external time-harmonic-driving force

**DOI:** 10.1038/srep11278

**Published:** 2015-06-10

**Authors:** Jinyong Ma, Cai You, Liu-Gang Si, Hao Xiong, Jiahua Li, Xiaoxue Yang, Ying Wu

**Affiliations:** 1 Wuhan National Laboratory for Optoelectronics and School of Physics, Huazhong University of Science and Technology, Wuhan 430074, China; 2 Key Laboratory of Fundamental Physical Quantities Measurement of Ministry of Education, Wuhan 430074, China

## Abstract

We propose a potentially valuable scheme to measure the properties of an external time-harmonic-driving force with frequency *ω* via investigating its interaction with the combination of a pump field and a probe field in a generic optomechanical system. We show that the spectra of both the cavity field and output field in the configuration of optomechanically induced transparency are greatly modified by such an external force, leading to many interesting linear and non-linear effects, such as the asymmetric structure of absorption in the frequency domain and the antisymmetry breaking of dispersion near *ω* = *ω*_*m*_. Furthermore, we find that our scheme can be used to measure the initial phase of the external force. More importantly, this setup may eliminate the negative impact of thermal noise on the measurement of the weak external force in virtue of the process of interference between the probe field and the external force. Finally, we show that our configuration can be employed to improve the measurement resolution of the radiation force produced by a weak ultrasonic wave.

The optomechanical system plays a significant role in the manipulation of mechanical resonators and electromagnetic fields, and leads to extensive topics, such as gravitational-wave detectors^1^,^2^,^3^, cooling of mechanical resonators^4^,^5^,^6^,^7^,^8^,^9^,^10^, single-photon transport^11^, optomechanically induced transparency(OMIT)^12^,^13^,^14^,^15^,^16^,^17^, and some other meaningful investigations^18^,^19^,^20^,^21^,^22^,^23^,^24^,^43^,^44^,^45^. OMIT is an analog of electromagnetically induced transparency(EIT)^25^,^26^,^27^,^28^, which provides an effective approach of controlling electromagnetic fields and the optical characteristics of matter. This emerging subject has led to many meaningful applications, including experimental ones (such as slow light^14^,^16^,^29^) and theoretical ones (such as enhance Kerr nonlinearities^30^, single-photon router^31^, and the precision measurement of phonon number^32^, coupling-rate^33^ and electrical charge^34^). More notably, second- and higher-order sidebands, which have been theoretically predicted and observed in a generic optomechanical system^35^,^36^, may lead to nonlinear versions of OMIT, which provides a new way of measuring the mechanical oscillator^37^,^38^.

The optomechanical system is composed of an optical cavity, in which one mirror is fixed while the other one is movable (it is treated as a mechanical resonator with frequency *ω*_*m*_), as shown in Fig. 1. The presence of a control field (pump field) and a probe field induces an anti-Stokes field produced from the scattering of light from the control field, which interferes with the intracavity probe field, leading to the transparent window in the spectrum of output field^12^,^13^. In this work, we impose an external time-harmonic-driving force, with amplitude *A*, frequency *ω* and initial phase *ϕ*_1_, to the moving mirror in the system. Such a driving force can be achieved through many ways, such as optical beat^13^, time-varying charges^34^ or current, and many other time-harmonic forces. The external force we introduce here is completely different from the force induced by intracavity fields since its properties cannot be affected by both the cavity and the mechanical resonator. But such an external driving force is able to induce the anti-Stokes fields excited from pump field as well, which is capable of modifying the output field significantly. Therefore, the output field can be used to probe the behaviors of a weak external time-harmonic-driving force. For example, the configuration in Fig. 1 is employed to measure the properties of a time-harmonic current. The interaction between the two parallel conductors with an unknown time-harmonic current generates a time-harmonic force to the movable mirror, and the properties of this force are obtained from the analysis of the output field. Then, we can obtain the properties of the time-harmonic current from the correlation between the force and the current. With the similar method, this setup is able to be employed for probing the features of an unknown ultrasonic wave or some other things which can exert external force on the movable mirror.

A previous work^39^ discussed the modifications of OMIT and optomechanically induced absorption in the presence of an external force. However, these effects are all the results of the linear interaction between intracavity probe field and external force. In this paper, we further discuss the effects within first-order process, particularly present the non-linear effects of the second-order sideband, and clearly point out the important potential applications hidden behind our configuration. In the first-order process, we find that there are asymmetric structures in the absorption spectrum of the cavity due to the modification of the external force, meanwhile antisymmetry breaking of dispersion near *ω* = *ω*_*m*_ because of the same reason. Moreover, these structures can be tuned by the phase difference between the probe field and the external force since such phase difference directly contributes to the phases of the intracavity fields, and therefore affects the interferences process of the intracavity fields. Then we show that the output field can be modulated by the external force in both first- and second-order processes. Within the approximations made in our work, we would like to point out that the effects induced by radiation pressure and external force are linear in the first-order process but non-linear in the second-order process. Thus, we are able to measure the initial phase of the external force through analyzing the impact of it on the output field, which cannot be achieved in the traditional measuring setup that probe laser is absent. Further, thermal noise may be neglected in our configuration, which may greatly contribute to the precision measurement of the external force. Finally, we find that our setup can be used to improve measurement resolution of the radiation force produced by a weak ultrasonic wave.

## Results

### Optomechanical system in the presence of external-force

**Figure 1:**
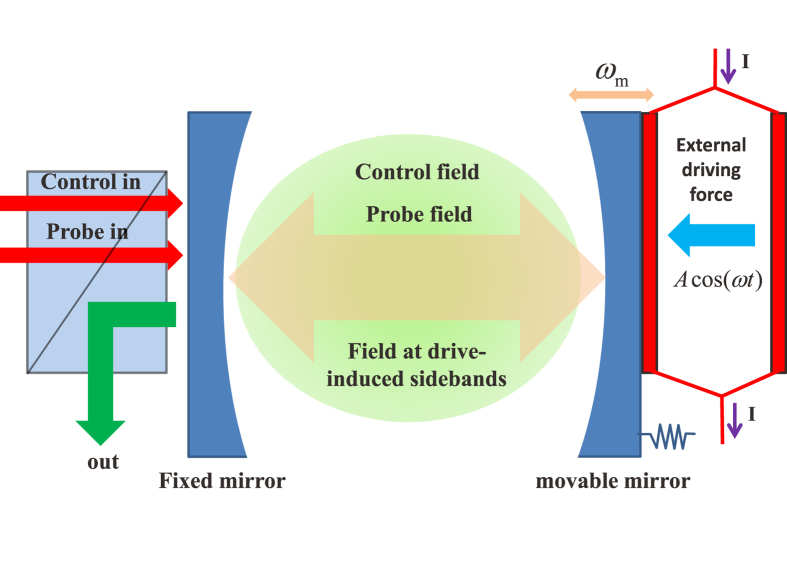
Schematic diagram of the optomechanical system with an external driving force. The system is composed of an optical high-frequency cavity, in which one of the mirrors is fixed but another one is movable with a low frequency *ω*_*m*_ and high Q. It is driven by a strong control field with frequency *ω*_*l*_, a weak probe field with frequency *ω*_*p*_, and an external driving force with frequency *ω*, amplitude *A* and initial phase *ϕ*_1_. The external force induces a serial of novel phenomena.

We describe an optomechanical system in the presence of a time-harmonic-driving force (see Fig. 1) by considering the Hamiltonian as follows:


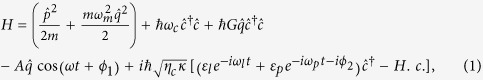


where *m* is the effective mass of the movable mirror with eigenfrequency *ω*_*m*_, and the 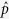 and 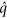 are, respectively, its momentum and position operator. The second term describes the cavity field and the term 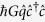 is for the coupling between movable mirror and cavity field. The term 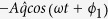 represents the external driving force we phenomenologically applied to the movable mirror, where *A* is the amplitude of the time-harmonic-driving force with frequency *ω* and initial phase *ϕ*_1_. The last term 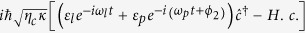, where *ϕ*_2_ is the initial phase of the probe field, is for the input field, which includes a weak probe field with amplitude 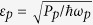 and a strong control field with amplitude 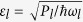, where *P*_*p*_ and *P*_*l*_ are the power of the probe field and control field, respectively. *κ*, the total loss rate, consists of an external loss rate *κ*_*ex*_ and an intrinsic loss rate *κ*_0_. *η*_*c*_ = *κ*_*ex*_/(*κ*_*ex*_ + *κ*_0_) denotes the coupling parameter^13^ and we choose *η*_*c*_ = 1/2 in this work.

### First- and second-order sidebands induced by probe field and external force

In a frame rotating at *ω*_*l*_, we define Δ = *ω*_*l*_ − *ω*_*c*_, *δ* = *ω*_*p*_ − *ω*_*l*_, and consider the decay rate *γ*_*m*_ of the movable mirror. Also, we consider the quantum and thermal noise of the mechanical oscillator 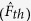 and cavity 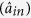. In the present work, we are interested in the mean response of this system; therefore we can analyze the expectation values instead of the operators. From 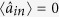 and 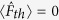, we take account of the first-order sidebands and ignore the higher ones at first, viz. using the following ansatz:


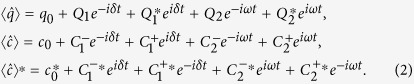


We obtain 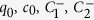 by using perturbation method as follows:


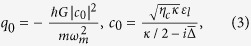



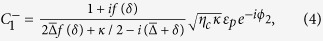



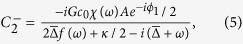


where


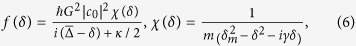



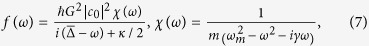


with 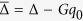.

Equation (3) describes the steady case without the probe field and the external driving force. If the control field is strong enough, the bistability of this system will appear. Therefore, our work focuses on the area of monostabillity^35^. Otherwise, Eq. (4) is the same as the previous work^13^, which systematically studied optomechanically induced transparency with this result. Moreover, Eq. (5) is used for discussing the effects induced by external driving force.

Then, we define 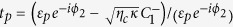, a dimensionless quantity, to characterize the behaviors of output field. This quantity also reflects the behavior of optomechanically induced transparency. It reads as^13^


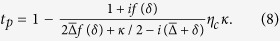


Further, when the frequency of the external force is detuned by zero from probe field, viz., *δ* = *ω*, the term 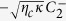 in Eq. (24) also describes the output field with frequency *ω*_*p*_. At this point, the dimensionless quantity to describe the features of output field under the action of external force reads:


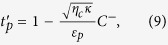


with


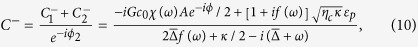


where *ϕ* = *ϕ*_1_ − *ϕ*_2_, the phase difference between the probe field and the external driving force. 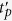 has the similar formation as the traditional OMIT, while it describes the combined effect of external force and control field.

For further discussion, we consider the process of the second-order sidebands affected by the external driving force in this optomechanical system. Then the 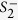, the amplitude of the second-order optical sideband in which we are interested, is obtained as follows:


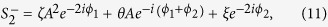


where,


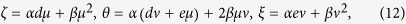



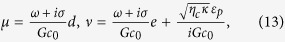



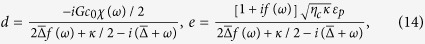



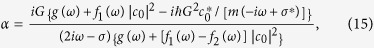



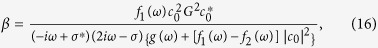



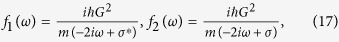



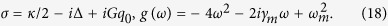


From Eq. (11), it is shown that the process of second-order sideband is modulated by both the external driving force and the probe field, and the effects induced by these two contributions are dependent and non-linear, which is different from the process of first-order sidebands. Without imposing the external driving force to the movable mirror, viz. *A* = 0, Eq. (11) is reduced to 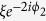, which describes the effects of second-order sideband in original optomechanical system^36^.

### Absorptive and dispersive behaviors of cavity

Let us analyze the effects of first-order sideband process at first, including the behaviors of absorption and dispersion of the cavity and the tunable properties of output field.

**Figure 2:**
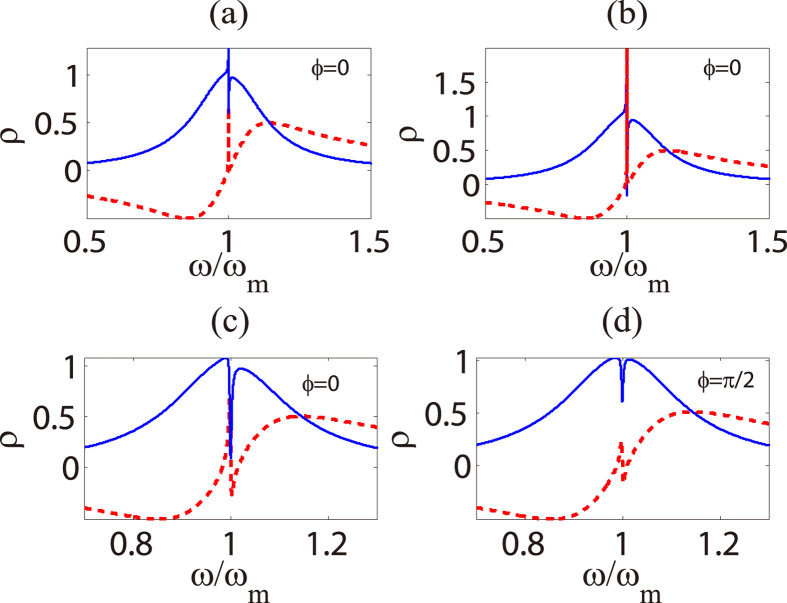
Absorption and dispersion of cavity. Calculation result of the absorption (solid blue curve) and dispersion (dashed red curve) of the cavity under the action of external driving force when *δ* = *ω* and *ϕ* = 0 by using Eq. (19). Setting the amplitude of external force *A* = 1.0 × 10^−9^*N* for panel (**a**), and *A* = 3.0 × 10^−9^*N* for panels (**b**–**d**). We use *P*_*l*_ = 1.49 *μ*W in panels (**a**,**b**), *P*_*l*_ = 456 *μ*W in panels (**c**,**d**). Other system parameters are taken as: *m* = 20 ng, *ω*_*m*_ = 2*π* × 51.8 MHz, *γ*_*m*_ = 2*π* × 41.0 kHz, *G* = −2*π* × 12 GHz/nm, *κ* = 2*π* × 15.0 MHz and Δ = −*ω*_*m*_, which are chosen from the recent experiment^13^. we fix *ε*_*p*_ = *ε*_*l*_/10 throughout the work since we should ensure that probe field is much weaker than control field, otherwise the perturbation method will become invalid and non-perturbative effects will occur^46^,^49^.

We define a dimensionless quantity


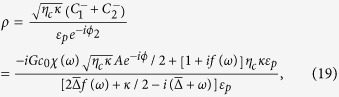


whose real quadrature shows the absorptive behavior of the cavity and imaginary quadrature describes the dispersive behavior. Figure 2, shows absorptive and dispersive behavior of the cavity under the action of external driving force when *δ* = *ω*. From Fig. 2(a), we find that the external driving force gives rise to the asymmetric structure of absorption and antisymmetry breaking of dispersion near *ω* = *ω*_*m*_. Furthermore, from Fig. 2(a,b), it is shown that the stronger amplitude makes the dip of absorption deeper and the peak of dispersion higher in the case of a weak control field. We should note that the probe power increases simultaneously when we enhance the control power since we fix *ε*_*p*_ = *ε*_*l*_/10. The optical pressure thus grows even faster, namely proportional to control power. We give some physical insight for these effects as follows: the radiation pressure exerted by probe field and the external driving force leads to anti-Stokes scattering of light from pump field, which produces two kinds of anti-Stokes fields induced by probe field and external force respectively; then the destructive interference of these two anti-Stokes fields and intracavity probe field results in such new phenomena. Otherwise, we find that both absorptive and dispersive spectrum cannot be extremely modified when *ω* is away from *ω*_*m*_, since the anti-Stokes field induced by external force is relatively weak in such case.

In addition, from Eqs (4) and (5), we are able to easily find that the phases of the probe-field-induced cavity field and the external-force-induced cavity field depend on the phases of the probe field and the external force respectively. The phase difference of the probe field and the external force therefore plays a significant role in the interference process between two different cavity fields, which means that it is an important parameter to modulate the total cavity field. Figure 2(c,d) show the absorptive and dispersive behaviors of the cavity by using different phase difference *ϕ* and pump power *P*_*l*_ in the case of identical amplitude of the external driving force. From Fig. 2(c,d), we find there is a deep dip near *ω* = *ω*_*m*_ and an asymmetric structure in the absorption spectrum as Fig. 2(c) when *ϕ* = 0, while the dip becomes shallow and the remarkable asymmetric structure disappears when *ϕ* is changed to *π*/2. In the process of interference, the field at traditional sidebands will be enhanced if it propagates in the cavity with the same phase as the field at the first-order sideband induced by external driving force, however, if the phase difference of these two fields is *π*, it will be weaken. Therefore, the phase *ϕ* can tune the absorption of the cavity.

### Output field modulated by external driving force in the first-order process

**Figure 3:**
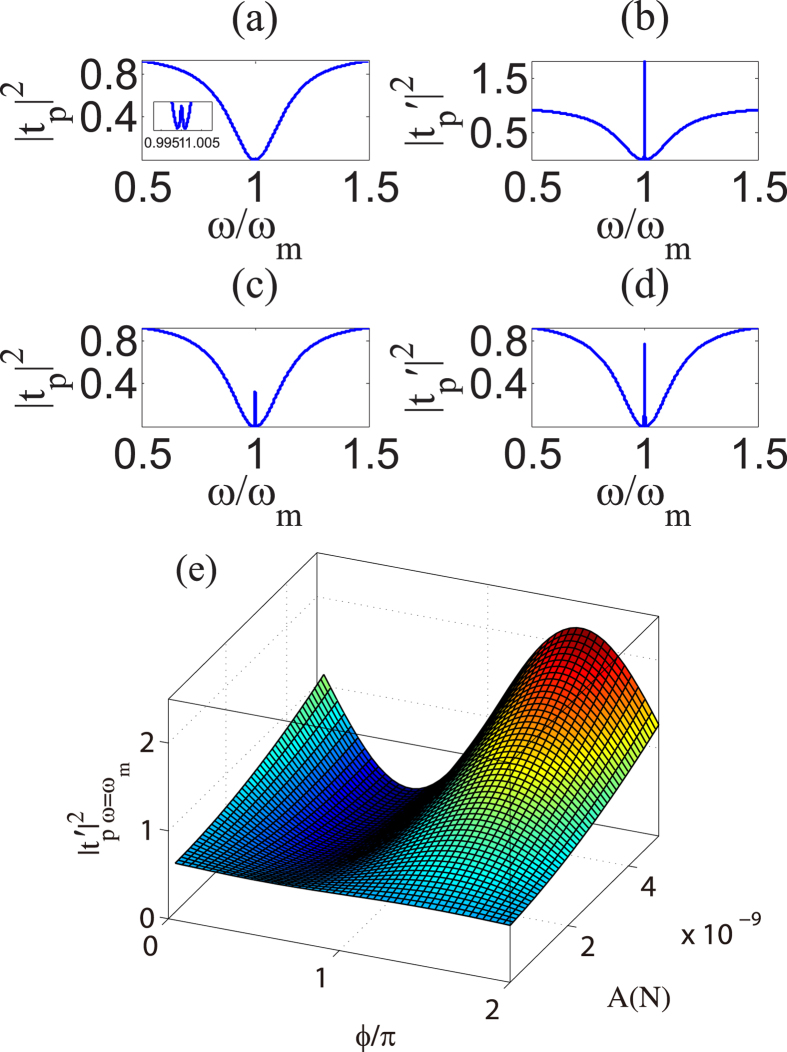
Modification of spectrum of output field in the presence of external force. Panels (**a**,**c**) correspond to different pump power *P*_*l*_ = 1.49 *μ*W and *P*_*l*_ = 0.149 mW respectively, and we use *A* = 0 for them. The figures on the right show the impact of external driving force on output field under the same external-force amplitude *A* = 2.0 × 10^−9^, and the pump powers of them are correspond to the figures on the left. Panel (**e**) is the calculation result of value of peak in the spectrum of output field varies with the phase *ϕ* and amplitude *A* of external driving force under the same intensity of control field *P*_*l*_ = 456 *μ*W.

In this subsection, we will discuss the impact of external driving force on the properties of output field. Figure 3(a,c) characterize the output field in the absence of external driving force, i.e., the generic OMIT signal^13^. The figures on the right in Fig. 3 by using *ϕ* = 0 show the impact of the external driving force on the output field under the same external-force amplitude *A* = 2.0 × 10^−9^*N*, and the pump powers of them correspond to the figures on the left in Fig. 3. From Fig. 3(a), it is shown that the control field can still induce a transparent window for the cavity near the resonance condition *ω* in the case of a quite weak control field, however, the OMIT signal is extremely faint. In this case, if we impose an external driving force to the movable mirror, the output field will be greatly modified by the external force, which is seen from Fig. 3(b). From Fig. 3(c,d), we find the external driving force enhances the peak value of transparent window. We consider similar physical process as previous subsection to explain this phenomenon. From the analysis of the intensity of external-force-induced sideband 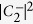 in the previous section, it is shown that when the external-force frequency *ω* is close to *ω*_*m*_, the mechanical motion driven through the external force induces a strong optical sideband field due to the anti-Stokes scattering of light, which intervenes with the probe field and the anti-Stokes field induced by probe field, leading to the modification of output field.

In addition, we have shown that the phase can tune the absorptive and dispersive properties of the cavity in the previous subsection. The significance of the phase difference between the external force and probe field is also reflected from the signatures of output field. Figure 3(e) describes that the peak value 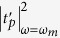 varies with the phase difference *ϕ* and external-force amplitude *A* under the same pump power. It is shown that 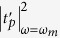 reaches the minimum near *ϕ* = *π*/2 and the maximum near *ϕ* = 3*π*/2 under the identical amplitude *A*. That is, the peak in the spectrum of output field is modulated by *ϕ*. Apparently, this phase-dependent effect can be explained by the process of interference. Interestingly, from Eq. (9), it is shown that 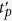 can easily exceeds one as long as *ε*_*p*_ is small enough, which can be seen from Fig. 3(e). Moreover, with the increase of external-force amplitude, the variation of 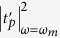 depending on *ϕ* is more sensitive. There are remarkable peak and valley when *A* is greater than 2 × 10^−9^*N*. Furthermore, we find that 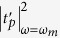 under the action of the external force near *ϕ* = *π*/2 is smaller than that without external force. That is, the presence of external driving force weakens the generic output field near *ϕ* = *π*/2. Hence, the phase difference between external force and probe field is an effective parameter to tune the output field.

### Output field modulated by external driving force in the second-order process

Up to now, we have shown some effects associated with the first-order sidebands induced by external driving force in the previous subsections. Even though it is shown that the amplitude and phase of the external force can tune the probe field effectively due to the process of interference, the discussions about these effects are based on the sum of two independent equations, Eqs (4) and (5). However, the second-order sidebands show some non-linear effects. From Eqs (11, 12, 13, 14, 15, 16, 17, 18), it is shown that Eq. (11) is reduced to 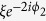 without external driving force and 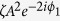 without probe field. Therefore, the term 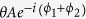 in Eq. (11) describes the non-linear interaction between the external driving force and the probe field.

**Figure 4:**
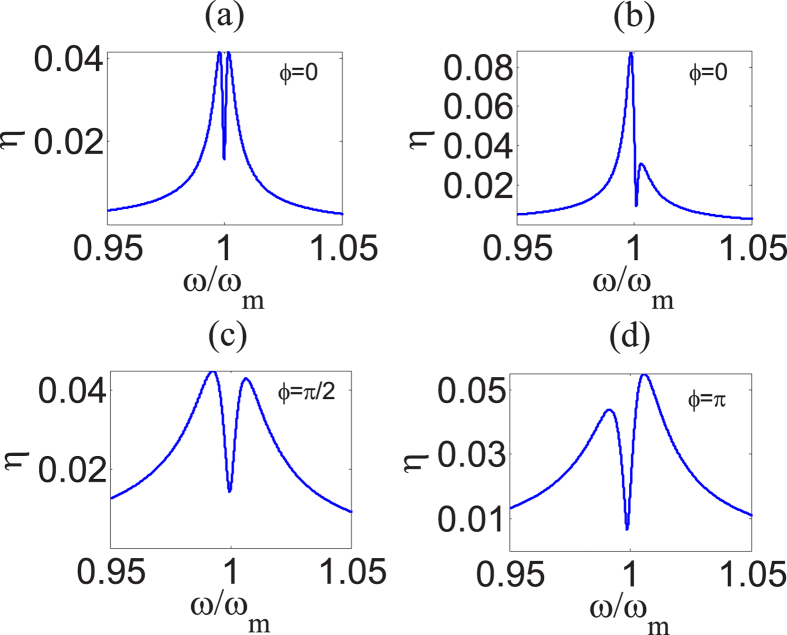
Efficiency of second-order sideband process. Calculation result of *η* varies with *ω* under the same phase difference *ϕ* = 0. We use *A* = 4 × 10^−9^*N* in panels (**b**), and *A* = 0 in panels (**a**) and. We consider the optical power of control field *P*_*l*_ = 456 *μ*W in panels (**a**) and (**b**). Also, *P*_*l*_ = 1.8 mW and *A* = 4 × 10^−9^*N* are used in panels (**c**,**d**).

In order to discuss the effects induced by second-order sidebands in this optomechanical system, we define.


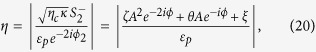


which is a dimensionless quantity to describe the efficiency of second-order sideband process. Figure 4(a) shows the case without external driving force and Fig. 4(b) describes the effect under the external-force amplitude *A* = 4 × 10^−9^*N*. There is a dip near *ω* = *ω*_*m*_, which was explained in ref.^37^. It is shown that the external driving force also leads to a similar obvious asymmetric structure in the process of second-order sideband as the effect of first-order sideband, which is the result of the upconverted first-order sideband process. The structure that the left peak is higher than the right one is shown in Fig. 4(b) when *ϕ* = 0. Therefore, the the impact of upconverted process of first-order sideband on the right peak is weaker, compared with that of left peak. Moreover, apparently, the external driving force can effectively enhance the efficiency of second-order sideband process.

In the previous subsections, we show that the phase *ϕ* makes a great contribution to the effects associated with the first-order sidebands due to the process of interference. The phase *ϕ* also plays a significant role in the second-order process. Figure 4(c,d) show that *η* varies with *ω* under different *ϕ*. We find that *ϕ* can tune the values of the two peaks and the depth of the dip. Moreover, the dip is shallower when *ϕ* = *π*/2 [see Fig. 4(c)] as a result of the upconverted first-order sideband process in comparison with the case of *ϕ* = *π* [see Fig. 4(d)], because the similar effect is found in the description of the real quadrature of *ρ* in Fig. 2(d).

**Figure 5:**
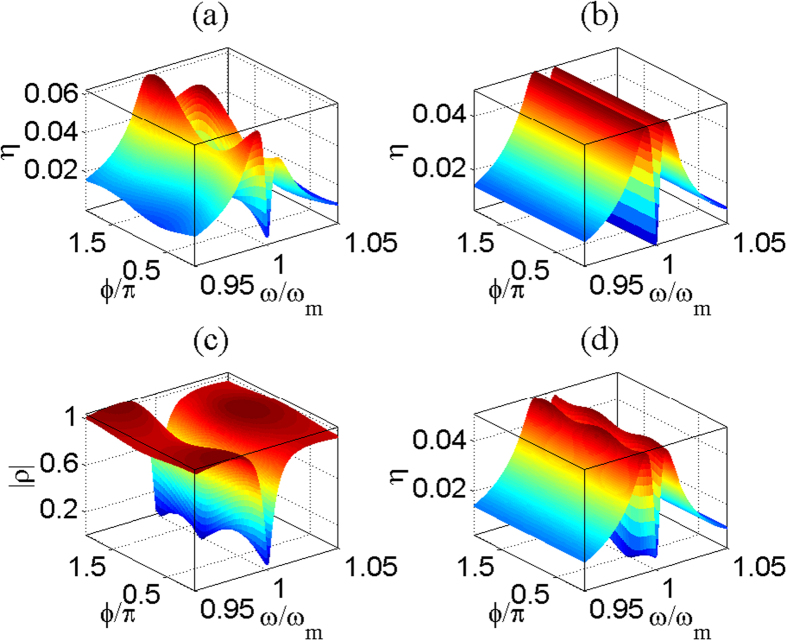
Phase-dependent effects of output field in the second-order process. Calculation result of *η* and the 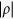 vary with *ϕ* and *ω*. We use *A* = 4 × 10^−9^*N* and *P*_*l*_ = 1.8 mW in panels (**a**,**c**,**d**). We take account of the case without external driving force under the same intensity of control field *P*_*l*_ = 1.8 mW in panel (**b**).

**Figure 6:**
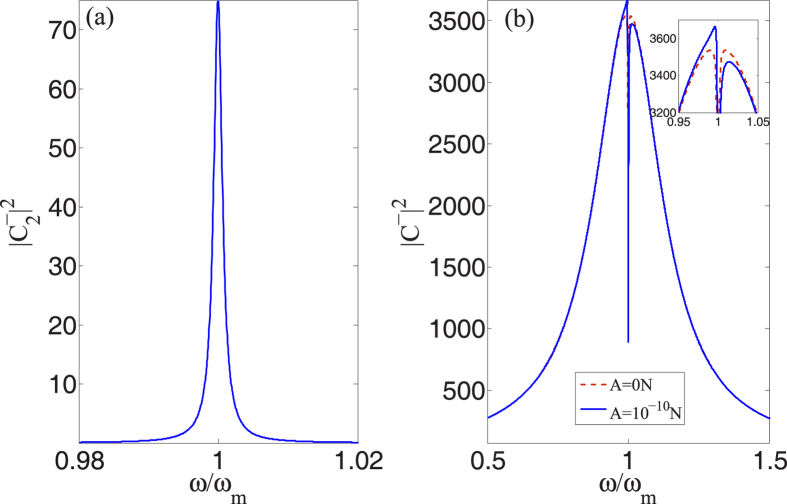
Scheme that may eliminate thermal noise. We consider the intensity of cavity field in the absence of probe laser in panel (**a**) and the interference of external force and probe field in panel (**b**). We use *A* = 10^−10^*N*, *ϕ* = 0 and *P*_*l*_ = 0.1 mW, and set *ε*_*p*_ = *ε*_*l*_/40 in this figure.

In order to better illustrate the upconverted first-order sideband process and the non-linear effect, the processes that describe that *η* and 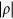 vary with *ϕ* and *ω* are shown in Fig. 5. We use *A* = 4 × 10^−9^*N* and *P*_*l*_ = 1.8 mW in Figs 5(a,c) and 6(d), and consider the case without external driving force under the same pump power in Fig. 5(b). From Fig. 5(a) and 5(b), we find that the external driving force leads to the phase-dependent effect. Figure 5(c) describes the first-order process, which indicates that the external driving force makes more contribution to the second-order process than the first-order one even if the effect induced by second-order sideband is due to upconverted first-order sideband process. Figure 5(d) is obtained from Eq. (20) by ignoring the term *θAe*^−*iϕ*^, which means it describes the simple sum of the two contributions of the external driving force and probe field. Therefore, Fig. 5(a) shows the non-linear interaction between the external force and probe field. In view of these discussions, the process of second-order sideband can be modulated by an external driving force, or provides an effective method to probe the features of an unknown time-harmonic force.

### Measurement of phase and amplitude of external force

In some previous works^47^,^50^, the weak-external force was measured by directly detecting the sideband induced by itself. For one thing, we cannot obtain the initial phase of the external force with this method. More importantly, the sideband induced by the external force is overwhelmed by the one induced by thermal noise when the force become extremely weak. However, in our configuration, i.e., in the presence of probe field, we are able to effectively measure the initial phase of an time-harmonic-driving force and may eliminate the impact of thermal noise on the measurement of the external-force amplitude.

Let us look into the measurement of phase first. Even though we could theoretically obtain the phase of external force from Eq. (24) in the absence of probe laser, it is pretty difficult to detect the phase of a single optical field without interference in the experiment. As a probe laser joins in, the field induced by the external force can interfere with the intracavity probe field. Then we can measure the relative phase of them by using the process of interference [see Eq. (19)] or the non-linear effect of the second-order sideband [see Eq. (20)]. Therefore, we are capable of obtaining the phase of the unknown time-harmonic force with our device.

In addition, we investigate another benefit of our configuration. We take account of a relatively weak external force (*A* = 10^−10^*N*) in Fig. 6. In such case, the field induced by the external force is pretty weak as well [Fig. 6(a)], and hence the impact of thermal force may have to be considered in the traditional setup^40^,^41^. Fortunately, the average phase of thermal noise is zero, which indicates that the field induced by the thermal noise cannot interfere with intracavity probe field, but the field induced by the external force can. Thus, by using the process of interference, we may use probe field to pull the field induced by the external force out of the region where is overwhelmed by thermal noise, which can be seen from Fig. 6(b). From Fig. 6(b), we find that the external force just changes the top of the curve where cannot be affected by thermal noise. Thereupon we may precisely obtain the amplitude of the external force by measuring the variation of the intensity of output field [see Eq. (9)] after the unknown external force is exerted on the mechanical resonator (the small change of the intensity of optical field can be easily detected). Admittedly, the accurate and convinced results have to be substantiated by following quantum theory and considering the thermal noise in the calculation.

On the other hand, we find this configuration can be used for precision measurement of ultrasonic radiation force. Ultrasound receives more and more attention in recent years. And the measurement of ultrasonic power plays an quite significant role in the assessment of ultrasonic equipment. Some previous works^40^,^41^,^48^ used the method of radiation force balance^44^ to measure ultrasonic power. The measurement resolution of radiation force produced by measured ultrasound is 10^−8^*N*^48^, which limits the detection of weak ultrasonic wave. But we are capable of using the configuration introduced in this paper to measure the power of weak ultrasound because the ultrasound that we need detect can produce time-harmonic radiation force exerted on the movable mirror [See Fig. 1]. From the discussion of Fig. 6, we find that we may measure the weak radiation force whose amplitude is as low as 10^−8^*N*, which extremely improves the measurement sensitivity of radiation force. Since an experimental work^42^ already has demonstrated force sensitivities down to 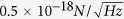 with an optomechanical system, the measurement sensitivity of ultrasound may be improved by using other experimental parameters or adding some other setups.

## Discussion

In this paper, we have investigated some combined effects of a pump field, a probe field and an external driving force in an optomechanical system, which are reflected in absorptive and dispersive behaviors of the cavity, and in the tunable features of output field both in first- and second-orders sidebands processes. In the first-order process, we find that there is an asymmetric structure in the absorption of the cavity even though the control field is extremely weak, and antisymmetry breaking of dispersion near *ω* = *ω*_*m*_ in frequency domain. Moreover, we find these structures are modulated by not only external-force amplitude but also its phase. Furthermore, we showed that the amplitude and initial phase of the external force are able to modify the spectrum of output field in the first process due to the destructive interference of the intracavity probe field and the anti-Stokes fields induced by external force and probe field, and in the and second-order process owing to the upconverted non-linear process. Therefore, we provide two potentially effective methods to precisely measure the amplitude and phase of an external time-harmonic-driving force: In the first method, we can probe the amplitude of the external force in the absence of probe field at first[see Eq. (5)], and then measure the phase of external force in the presence of probe field by using Eq. (9). Alternatively, we can combine the first- and second-order processes by employing Eq. (9) and Eq. (20) to measure the amplitude and phase of the external force simultaneously. More importantly, we find that our configuration may eliminate the negative impact of thermal noise on measurement and be employed to detect weak ultrasonic radiation force.

### Derivation of mechanical and optical sidebands in the presence of external force

We obtain the Heisenberg-Langevin equations from Eq. (1) as follows


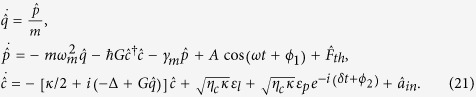


In this work, we consider the mean response of this system, Eq. (21) becomes


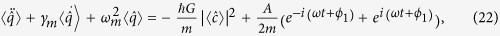



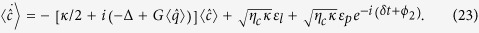


Equations (22) and (23) are non-linear, and we can use perturbation method to obtain approximate analytical solution for the case that the probe field is much weaker than the pump field. The solution is composed of the terms with different frequencies, while here we consider the first-order sidebands. By plugging the ansatz Eq. (2) into Eqs (22) and (23), and retaining the first-order terms, we can obtain 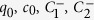.

The output field can be obtained by using the input-output relation:


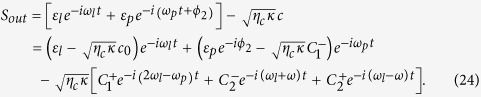


From Eq. (24), it is shown that the terms 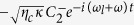 and 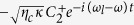 describe the sidebands induced by external driving force, which will lead to a serial of effects. In this paper, we take account of the sideband with frequency *ω*_*l*_ + *ω* (external-force-induced sideband) and define 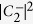 as the intensity of this sideband.

Then we consider the process of the second-order sidebands affected by the external driving force in this optomechanical system. Due to the non-linear terms 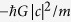 in Eq. (22) and −*iGqc* in Eq. (23), we make another ansatz with higher-order terms when *δ* = *ω*:


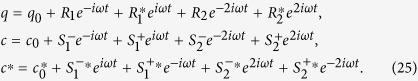


We solve Eqs. (22) and (23) by using ansatz (25) and take account of such a second-order process that the amplitude of probe field is much stronger than the one of second-order sidebands. Then we are able to obtain the amplitude of the second-order optical sideband 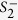.

## Additional Information

**How to cite this article**: Ma, J. *et al.* Optomechanically induced transparency in the presence of an external time-harmonic-driving force. *Sci. Rep.* **5**, 11278; doi: 10.1038/srep11278 (2015).
